# Parasites

**Published:** 1892-04

**Authors:** Cooper Curtice

**Affiliations:** Veterinarian, Moravia, New York


### PARASITES.

### BEING A LIST OF THOSE INFESTING THE DOMESTICATED ANIMALS AND MAN IN THE UNITED STATES.

---

By Cooper Curtice, M. D., Veterinarian, Moravia, New York.

---

There is soon to appear the second edition of Neumann's "Traite des Maladies Parasitaires" and its English translation by Flemming. This book is by all odds the best yet printed on this subject for the veterinarian in general practice, and either the original or its translation should be in the hands of each practitioner. The book is, of course, written primarily for the French student, but so broad is its treatment of the subject that when translated, its use is cosmopolitan.

In the "Animal Parasites of Sheep," issued by the Department of Agriculture, if it was shown that of the nineteen species of parasites found in and on sheep in this country, but two were indigenous, viz.: *Tænia fimbriata* and *Oesophagostoma columbianum.* Of these the latter was assumed to be indigenous because it had not been described in Europe. Now, if we assume that the same ratios exist between the indigenous and introduced parasites of the other domesticated animals as between those of sheep, or admitting the presence of even a higher proportion of the indigenous species in the former, there will still be a very large proportion of all the parasites, which are common to the old world and this. That we have any indigenous parasites at all seems the surprising condition of affairs, when one considers the very few opportunities our domesticated animals have of acquiring parasites from other animals than themselves, and the present condition of each parasite which must have consumed such immensurable time in being arrived at.

Aside from "Verrill's External and Internal Parasites of Man and Domestic Animals, and Supplement," printed in 1870 by the Connecticut Board of Agriculture, there has been published no systematic list of the parasites of this country. In reply to the question whether he had observed all the species mentioned in these lists as occurring in this country, Professor Verrill replied that he had seen the majority and those not observed might be expected to be found here sooner or later. The lists while excellent are therefore not thoroughly reliable as showing distribution. The late Professor Leidy has also recorded a few of the species that he found in addition to those described by him: *Prof. Phil. Scud. Nat. Sci. 1856.* Other partial lists have been made from time to time by various writers, as occasion has offered, but none of them have attempted any degree of completeness.

In the succeeding lines it is my purpose to present a list of the parasites that I have found and collected in this country, together with some species which other observers have identified as occurring here. These lists are made from memory. They can, however, all be verified by the collections of the Bureau of Animal Industry or by published articles. Being made from memory and without consulting all the literature necessary to present complete lists of all the parasites which have been positively identified in this country they will be more or less incomplete. They are therefore presented as work accomplished and as affording working data for comparison with those presented by Neumann. They will also serve as a means of identification of the parasites found until the more complete hand-books on the animal parasites of cattle, horses, swine, dogs and poultry, to be issued by the Bureau of Animal Industry shall appear. If the student will study Neumann's work alone or in conjunction with "Verrill's Parasites", the "Animal Parasites of Sheep" and these lists, he will be able to make some headway in the determination of any species he may find.

The plan followed is essentially that of Neumann, who considers the different animals in turn subdividing the subject according to the various organs of that animal. It was the plan followed in "Parasites of Sheep" with tolerable convenience. So constantly does each parasite invade its appropriate organ, so rarely it is that a parasite is found in more than one animal, and so unfrequently are two species closely resembling each other found in the same organ, that the finding of a parasite in a given animal and organ goes far toward its determination. Presentation of the subject on this plan therefore affords a rough key to the determination of the species.

If there is no other comment, it is to be understood that I have seen the parasites, and that prior to April, 1891. My acknowledgements are due to many friends who have in past years sent me specimens, to Dr. Stiles, of Washington, D. C., who has loaned me books not otherwise available, and to Dr. F. L. Kilborne, of that city, with whom I have spent many pleasant hours in collecting, for the use of his private collection.

SHEEP.

Skin: *Melophagus ovinus* Linn., sheep ticks. *Trichodectes sphærocephalus* Nitzsch. The sheep-lice are found more abundantly on the inside of the fore legs. *Sarcoptes scabiei* de Geer var. *ovis*, head scab mites; identified from newspaper descriptions. *Psoroptes communis* Fürst var. *ovis*, common scab mites.

Nose: *Oestrus ovis* Linn., grubs in the head.

Diverse organs: *Tænia marginata*, Batsch., bladder worms in the omentum, liver, etc.

Stomach: *Strongylus contortus* Rud., twisted round worms; occur in the fourth stomach. *Strongylus* Ostertagi, (Ostertag) Stiles, reported personally by Dr. Stiles.

Duodenum and liver: *Tænia fimbriata* Diesing, the fringed tape worm. It also enters the gall and pancreatic ducts. *Distoma hepaticum* Linn., liver flukes; reported by sheepbooks and newspaper articles. This species seems to be rare in this country.

Small intestine: *Tænia expansa* Rud., the broad tape worm; occurs in the upper half of the middle. *Strongylus filicollis* Rud. and *Strongylus ventricosus* Rud. (?) (This species is queried because of certain minute differences between it and *S. ventricosus* of cattle;) minute round worms in the upper half of the intestine. *Ascaris lumbricoides* Linn., the large round worm in the jejunum; very rare in sheep. *Dochimius cernuus* Creplin, the bent head round worm, occurs scattered along the intestinal walls; produces minute hemorrhagic dots; usually found in animals recently slaughtered, attached to the intestinal wall; they feed on blood. *Coccidium perforans* (?) was found scattered in white patches along the intestine in but one case.

Large intestine: *Sclerostomum hypostomum* Duj., round-worm with a cup-shaped head. Found once in Colorado. *Oesophagostoma columbianum* Curtice, is found very abundantly in the mucous slime of the large intestine. The embryo is to be found in the cheesy tumors they make in the walls of the small and large intestines. *Trichocephalus affinis* Rud., the whip worm may be found sewed into the mucous membrane of the cæcum.

Lungs: *Strongylus ovis-pulmonalis* Diesing, the hair-lung worm. Found in lung tissue and the extreme ends of bronchial tubes. They cause the small tumors seen on the surface of many lungs. *Strong-*

*ylus filaria* Rud., the thread lung-worm is found in the bronchi toward the caudal tip of the lung. They cause plugging of the invaded bronchus and solidification of the corresponding lobule of lung.

Muscles: *Sarcocystis tenella* Rauillet, Sporozoa, recorded on authority of Dr. Stiles.

### CATTLE.

Skin: *Trichodectes scalaris* Nitzsch and *Hæmatopinus vituli*, Linn. Cattle lice. Professor Herbert Osborn in "Pediculi and Mallophaga," 1891 Rept. U. S. Dept. Agriculture records two species, *H. vituli* Linn., and *H. eurysternus* Nitzsch. I have collected forms from the same animal which could be assigned to each species, and some doubtfully to either. I prefer therefore to record but the one species. *Hæmatopinus macrocephalus* Burm., the horse louse, collected by Dr. F. L. Kilborne. *Demodex folliculorum*, recorded by W. Faxon, Bull. Comp. Zool., vol. V, 1878, pp. 11-17, *Böophilus bovis* (Riley) Curtice, the common cattle tick of the south, *Dermacentor Americanum* Linn., the dog tick of the north-east. The male of this species has two quite plain white lines running the length of his back, besides other smaller white spots; the female has her headshield white with a central dark spot. Each have the dorsum of the limbs white. *Dermacentor occidentalis* Marx. in MSS., the cattle tick of California. This cattle tick is like *D. Americanus*, but is somewhat smaller; the headshield of the female is decidedly whiter, the shield of the male being nearly white with small symmetrically situated dark spots toward either side. *Ambylomma unipunctata* Packard; the male of this species is characterized by two marrow iridescent longitudinal lines; the female by an iridescent spot at the caudal edge of her headshield. This species occurs from Maine to Texas. *Ixodes scapularis* Say. On cattle in New York. *Ixodes*, n. sp. from a cow in California. *Hypoderma lineata* Villers, the larvæ of which occur in skin tumors of the back; They are also found in the œsophagus, spinal column, muscles of the back, etc. *Lucilia macellaria* Fabr., this, the maggot of a fly, is the screw-worm of the southern United States, and may be considered as a parasite. It not only bores into the depth of wounds of the skin but has also been found by Dr. M. Francis in the fourth stomach of a calf. *Stomoxys calcitrans*, Geoff. and *Hæmatobia serrata* Rob. D. are the large and small biting-flies which become so annoying to cattle in midsummer. The former resembles the house-fly in size and appearance. The latter has been called "horn-fly" which is not a good name.

Oesophagus: *Myzomimus scutatus* (Müller) Stiles, is a hair-like worm occurring quite abundantly in the eastern United States. It is found beneath the epithelial tissue woven into the mucous coat by pretty, uniform loops which taken together present a ladder-like appearance. This species was first observed in this country by Dr. Theobald Smith of the Bureau of Animal Industry while studying the "Southern cattle fever" in November, 1889. It has lately been assigned to a new genus, *Myzomimus*, created for it by Dr. C. W. Stiles now in charge of the studies on animal parasites in the Bureau of Animal Industry. See this Journal, February, 1892.

Stomach: *Strongylus contortus* Rud., a larger variety of this species than that in sheep occurs in the fourth stomach. *Strongylus Ostertag*, Stiles, 1892, (*Strongylus convolutus* Ostertag, 1891,) a minute round worm is also found, often associated with the above. It causes minute dot-like tumors which are scattered over the surface of the mucous membrane. This species was first seen in the Fall of 1890 by Dr. Smith. It seems to vary in size from the European described form, but after laying aside the incorrectness of Ostertag's description it will be difficult to separate the American form as a distinct species.

Duodenum and liver: *Distoma*; a large species of fluke, is found in the liver and lungs of cattle in the west and southwest. The species has been noted several times, and but lately described by Dr. A. Hassell, "Am. Vet. Review," July, 1891, and by Dr. M. Francis, Bulletin of Texas State Agricultural College, October, 1891. It is doubtful if either of the descriptions will hold over earlier European descriptions of *D. magnum*, Bassi, a similarly appearing parasite. For occurrence, see Flukes in Cattle, by Cooper Curtice, "Am. Vet. Review," page 390, 1887, and "Pacific Rural Press," January 24, 1871; *Echinococcus polymorphus* Diesing.

Small intestine: *Ascaris sp.* (?) A large ascaris is reported by butchers as being occasionally found. *Dohmius radiatus* Rud. occurs rather sparsely attached to the mucous walls; it is easily marked by its dark appearance; *Strongylus ventricosus*, Rud , very minute, but comparatively abundant in the jejunum. *Tænia expansa*, Rud , occurs in the middle of the small intestine *Tænia denticulata*, Rud., also occurs in the middle of the small intestine. It can be diagnosed from the former by the undulate appearance of the caudal margin of the adult proglottids, which also are rather longer than those of *Tænia expansa*.

Large intestine: *Trichocephalus affinis*, Rud., is found attached to the cæcal walls. *Oesophagostoma inflatum*, Schneider, (?) occurs in its adult state in the mucous slime, chiefly near the constriction below the cæcal enlargement. The embryos are found in the shot-like tumors of the walls of the small and large intestine. The presence of the worm inside easily characterizes them. In the European species (*O. inflatum*) the head is immediately followed by a transparent inflation of the skin. In the American species this inflation is divided into two nearly equal parts by a cincture. This difference being constant, our species is certainly varietal, perhaps specific. Not having had European forms for further comparison, I can not say whether this variation is accompanied by others or not.

Lungs: *Strongylus micrurus*, Mehlis, occurs in the bronchi. The worms, together with mucus and debris, plug the bronchi and produce consolidation of the affected part of the lung. Distoma, as noted above, has also been found. It occurred in hard nodules, in the center of the lung.

Abdomen: *Filaria cervina*, Duj. A form which occurs rather abundantly in the United States is referred, by the advice of Dr. Stiles to this species, and not to *Filaria papillosa*. It apparently floats free in the cavity. Both sexes and but few individuals of either may be found. *Tænia marginata*, Batsch, found in cystic stage in the mesenteries.

Eye ball: *Filaria cervina*, Duj. This worm has been seen by farmers in the anterior chamber of the eye.

Muscles: *Tænia saginata*, Goeze, occurs scattered through the muscles in its cystic stage. I have not yet seen a single specimen. Of the considerable number of adult tape-worms of man that I have seen, however, but three or four have been anything else, so one must conclude that this tape-worm must be fairly abundant in cattle. *Sarcosporidium sp.* On authority of Drs. Smith and Stiles; found in heart and other muscles.

Spleen: *Filaria lienalis*, Stiles, *Ms.*; a very long, weak form of thread worm was first found by Dr. Smith, August, 1890. The specimens had threaded themselves into the capsule of the spleen. I have seen but one other similarly located.

### HORSE.

Skin: *Trichodectes parumpilosus*, Piaget and *Hæmatopinus macrocephalus* Burm, horse lice. *Hypoderma lineata*, Villers grubs described in tumors of the back, by Texas ranchmen. *Amblyomma*

*unipunctata*, Packard, described to me personally by Dr. M. Francis. A species of *Argas* occurs in horses' ears in California. Specimens from Professor E. J. Wickson, Berkely, Cal. Other ticks may be expected. *Stomoxys culcitraus*, Geoff.

Stomach : *Gastrophilus equi* Fabr.; the larval stages are known as bots, and are found attached to the wall of fundus of stomach. *Spiroptera megastoma* Rud., a round worm occurs in tumors of the pyloric end. *Spiroptera microstoma*, Schneider ; I believe that I found this species once only.

Duodenum : *Gastrophilus nasalis* L. ; the larval stage is known as the bot, and is to be found attached to the wall. When fresh it is smaller than the *G. equi*, and is a pale green, while *G. equi* is red. *G. equi* carries a double row of spines on nearly every segment, while *G. nasalis* carries but a single row. In the adult the wings of *G. nasalis* are unspotted toward the tips, in *G. equi* they are spotted. *G. equi* is the larger.

Small intestine : *Ascaris megalocephala*, Cloquet, usually occur in the ileum. It is the largest of the round worms. *Tænia perfoliata*, Goeze ; specimens in the collection of Dr. R. S. Huidekoper. A very short and broad tapeworm.

Large intestine : *Oxyuris curvula*, Rud., white pin worms ; never found attached, always in the mass of the food. *Sclerostomun armatun*, Rud., dark pin worms ; usually found attached to the walls of the colon ; the young are found in tumors ; they also penetrate into the arteries and cause aneurisms. The adults may be picked out by their large, thick bodies and cup-like heads. *Sclerostomun ? tctracanthum*, Diesing, small, dark-pin worms. Very small worms usually found attached or near the mucous membrane. This and the preceding species may occur in the food mass. The young make tumors in the intestinal wall. The adults may be determined by the four terminal spines, which are quite prominent.

Abdomen : *Filaria equina* Abildgaard. Noted by castrators as occurring in the scrotum. Found once by myself in the abdomen. The name is used at the suggestion of Dr. Stiles. *F. papillosa* is a synonym.

### SWINE.

Skin: *Hæmatopinus suis*, L., the hog louse and *Lucilia macellaria*, Fabr., the screw worm are the only skin parasites that I have seen. Dr. Alfr. Dujes, of Guanauato, has described and noted *Argas turicata*, a species of tick.

Stomach : *Spiroptera strongylina*, Rud., is often found mixed with the food and attached to the walls. *Strongvlus rubidus*, Hassell and Styles, 1892, MS., a minute round worm but recently discovered.

Small intestine : *Ascaris lumbricoides*, Linn., very abundant in young pigs in the duodenum and jejunun. Often it pushes into the bile ducts and into the stomach. *Echinorhynchus gigas*, Goeze, may also be found in hogs which roam in the fields. It attaches by its spined proboscis to the intestinal walls and produces ulcers. It is easily distinguished from *Ascaris*, which is yellow, firm to the touch, and has a three-lipped head, by its white color, by its collapsing when pressed, into a flat worm, and by its spine head. *Trichina spiralis*, Owen, occurs in the adult state in the intestine during a transient period. I have not seen the adult. *Dochmius radiatus*, Rud. (?) Found by Dr. Kilborne in small intestine ; by myself, on one occasion only, in large intestine. The specimens that I have seen were immature, hence the doubt in regard to the specific name.

Large intestine : *Trichocephalus crenatus*, Rud., occurs attached to the cæcal wall. *Œsophagostoma dentatum*, Rud., usually concealed in mucous slime of the intestine.

Ureter : *Stephanurus dentatus*, Diesing, found once in cysts connected with the ureter; also found in the fat of the abdominal cavity by Professor Verrill, Dr. T. Smith and many others.

Abdomen : *Tænia murginata*, Batsch, cystic stage and *Stephanurus dentatus*, Diesing.

Muscles : *Trichina spiralis*, Owen, encysts in microscopic capsules. *Tænia solius*, Linn., cystic stage. Out of 1,037 swine examined by Osler and Clements,* 76 were infected. I have seen but three specimens submitted for examination. *Sarcocystis amiescheri* Lankester, a sarcosporidium in the muscles (Dr. Stiles).

Lungs : *Strongylus paradoxus*, Mehlis, invade the bronchi ; the species is similar in size and shape to the corresponding species in cattle and sheep, and cause a like disease.

Liver: *Tænia echinococcus*, v. Siebold, cystic stage; 31 animals infected in 1,037 examined by Clements and Osler, 1883. Dr. Wm. H. Welch,† Baltimore, Md., in a paper read March 17, 1890, recorded

* Parasites in the Pork Supply of Montreal, by Osler and Clements, 1883.

† Dr. Welch's list of parasites recorded in Johns Hopkins Hospital Bulletin, July, 1890, seems to be the first trustworthy work done in this direction in this country.

this parasite as found in pathological laboratory material. One case reported by Dr. V. A. Moore to the Washington, D. C., Biological Society, Nov. 14, 1891.

DOGS.

Skin: *Pulex serraticeps*, P. Gervais, the dog flea. *Trichodectes latus*, Nitzch, and *Hæmatopinus piliferus*, Burm, dog lice. Kilborne's collection. *Demodex folliculorum*, Owen, red mange mite. *Böophilus bovis* (Riley) Curtice, cattle tick found on a dog by Dr. Kilborne in Washington, D. C., and by Dr. Francesca de Balmaseda in Cuba. *Dermacentor americanus*, Linn., the common dog tick of the Eastern United States. *Ixodes scapularis*, Say, the tick common to dogs and small wild animals of the Eastern United States.

Nose: *Linguatula tœnioides*, Rud , presence indicated by the finding of eight specimens of this species in a rabbit by Dr. F. L. Kilborne, and one specimen at another time by myself.

Oesophagus: *Spiroptera sanguinolenta*, Schn., the round worms in tumors of the oesophageal wall.

Small intestine: *Ascaris marginata*, Rud., the large round worm; found often in stomach. *Dochmius trigonocephalus*, Rud., found scatteringly along the intestinal walls, characterized when alive by its dark-colored body. *Taenia echinococcus*, Von. Siebold, very minute; found but once in a dog killed at the Washington, D. C., pound. *Taenia marginata*, Batsch, in the middle of small intestine. *Taenia serrata*, Goeze, in the middle of small intestine. *Taenia coenurus*, Küch. The tape worms identified as *T. coenurus* were found but once in Colorado. The species may have been one arising from rabbit cysticerci and wrongly identified. The specimens were taken from a sheep dog and occurred in the ileum. They are now in the Bureau collection. *Taenia cucumerina*, Bloch, a small tape worm occurring in the ileum: very abundant. *Taenia serialis*, Baillet, presence in dogs in Texas and California, indicated by the cystic stage of this worm in the back muscles of jack rabbits.

Large intestine: *Trichocephalus depressiusculus*, Rud., in the cæcum attached to the walls.

Blood vessels: *Filaria immitis*, Leidy, in the right half of heart and the ascending vena cava; embryos in other blood vessels. Collection of Dr. Kilborne and Bureau.

Lungs: *Strongylus canis-bronchialis*, Osler, found by Dr. Osler and described in VETERINARIAN, July, 1877.

Kidneys: *Eustrongylus gigas*, Diesing, the larger kidney strongyle. Specimen No. 1,024, Army Medical Museum. Dr. William H. Welch records a specimen 95 cm. long, which was found free in the peritoneal cavity. He reports three other findings, Johns Hopkins Hospital Bulletin, July, 1890.

CATS

Skin: *Pulex serraticeps* P. Gervais, the cat flea. *Demodex folliculorum*, Owen, causes mange.

Intestine: *Ascaris mystax*, Rud., the large round worm of cats. *Tænia crassicollis*, Rud., the thick neck tape worm which develops from its cysticeren stage, found in mice and rats' livers. *Tænia elliptica*, Batsch., from the lower part of the small intestine. This species closely resembles, if it is not identical, with *Tænia cucumerina* of dogs. *Echinorhynchus campanulatus*, Diesing (?) found once by Dr. Kilborne. *Oxyuris compar.*, described by Prof. Leidy.

Muscles: *Trichina spiralis*, Owen, of which I have seen splendid specimens in Prof. S. H. Gage's laboratory at Cornell University, about 1880.

RABBITS; DOMESTIC. (*Lepus cuniculus*, Linn.)

Skin: *Cuterebra cuniculi*, Clark; grub lives in tumor under skin; *Hæmatopinus ventricosus*, D.; sucking lice of the body. *Sarcoptes notoedres*, Del. and Bourg, causes nose and foot mange. *Psoroptes cuniculi*, Megnin, causes excessive epithelial desquamation in the ears. *Böophilus bovis*, Riley, cattle ticks; in collection of Dr. Kilborne.

Intestine; *Taenia pectinata*, Goeze, the rabbit tape worm *Trichocephalus unguiculatus*, whip worm from the cæcum. *Oxyuris ambigua*, Rud., pin worm from the rectum.

Abdomen: *Taenia serrata*, Goeze, or *cysticercus pisiformis*, Zed., in the mesenteries. *Linguatula taenioides*, Rud., was found floating free in the cavity as heretofore stated.

Liver: *Coccidium oviforme* causes yellowish boil-like spots which appear on the surface of the liver.

RABBIT; WILD. (*Lepus Americanus*, Erxleben.)

Skin: *Cuterebra cuniculi*, Clark. *Haemaphysalis* sp. undt., the rabbit tick.

Intestine: *Strongylus retortaeformis*, Zed. (?) *Taenia pectinata*, Goeze. *Monostomum*, (?) sp. undt. *Trichocephalus unguiculatus*, Rud.

Abdomen: *Taenia serrata*, Goeze, cystic stage.

### OTHER RABBITS.

Nearly all wild rabbits harbor *cysticerci.* In Colorado, cotton tail rabbits (*Lepus sylvaticus*, Bachman) yielded in 1886 an undescribed *Taenia.* Jack rabbits (*Lepus texianus*, Waterhouse) still another species. Jack rabbits in Texas, (*L. texianus*), and California, *L. californicus*, Gray) are infested by a *coenurus.* These immature tape worms cause great swellings along the spinal columns. When cut into they are seen to be great sacs filled with smaller bladders and watery fluid. From hearsay, this tape worm must be prevalent in Virginia and southward in the larger rabbits. I have found a species of *Cuterebra* in jack rabbits of California, near Auburn.

### FOWLS.

External Parasites: *Menopon pallidun*, Nitzsch, body louse of hens. *Goniocotes Burnetti*, Packard, head louse of young chickens, collection of Dr. Kilborne. *Goniocotes hologaster*, Nitzsch, and *Goniocotes abdominalis*, Piaget, chicken lice seen by Osborn, "Fediculi and Mallophaga," p. 32. *Goniocotes compar*, Nitzsch, on pigeons. (Osborn), *Gonides stylifer*, Denny, turkey louse; *Lipeurus baculus*, Nitzsch; pigeon rod-louse (Osborn), *Lipeurus squalidus*, Nitzsch, squalid duck louse (Osborn) *Lipeurus variabilis*, Nitzsch, chicken red louse. *Ornithobius cygni*, Denny, white swan louse (Osborn). *Menopon stramineum*, Nitzsch, common hen louse. *Trinoton luridum*, Nitzsch, duck louse (Osborn). *Trinoton lituratum*, Nitzsch, goose louse (Osborn).

Internal Parasites: *Tænia infundibuliformis*, Duj., and *Tænia proglottina*, Dav. (?) tapeworms in chickens. *Echinorhynchus polymorphus*, Bremser, in ducks, *Heterakis inflexa*, Zeder, round worms in small intestine of hens. *Heterakis vesicularis*, Fröl., in cæca of fowls. *Heterakis maculosa*, Rud., small intestine of pigeons. *Syngamus tracheais*, von Siebold, the tapeworm of chickens. *Cytodites nudus*, Vizioli, mites found in the thorax, abdomen, and beneath the skin of hens. *Hypodectes columbarum*, mites in the body cavity of doves. *Distoma ovatum*, Rud., in hens' eggs, described by Dr. E. Linton, Proc. U. S. Nat. Museum, 1887, p. 367.

### GUINEA PIGS.

These animals are the freest from parasites of any species that I have examined. I do not remember having seen any internal parasite. The skin lice, *Gyropus gracilis*, Nitzsch, and *G. ovalis*, Nitzsch, have become quite abundant in the breeding pens of the

Burea of Animal Industry on one or two occasions. Although the type of each species seems quite distinct, there are many intermediate forms, if I remember rightly, which seem to bring them quite closely together. *Demodex folliculorum*, Owen, some guineas kept at the National Zoölogic Gardens presented all the appearances of the mange caused by these pests, February 22, 1892. No microscopic examination was made.

MAN.

Skin: *Dermatobia noxialis*, Goudot, a tumor-making fly-grub. Seen many times by others. An excellent account is given in Insect Life, vol. I, p. 76, 1888. *Hypoderma lineata*, Villers, also a tumor-making fly-grub. The larva wanders quite extensively under the skin before it finally begins its tunnel through the skin. See various articles in Insect Life by Riley and Howard, vols. I, II and III, U. S. Dep't. Agriculture, 1891. *Lucilia macellaria*, Fabr., the screw worm attacks open wounds and nasal chambers. See Insect Life, vol. II, p. 116, 1889, do. vol. IV, p. 159, 1891, and vol. IV, p. 200, 1891. *Pulex serraticeps*, P. Gervais. Dog fleas constitute quite a nuisance in our southern cities. *Sarcopsylla penetrans*, Linn., the chigoe of the south buries itself into the skin and produces pastules often reported. *Cimex lectularius*, Linn., bedbugs, chinches. *Pediculus capitis*, de Geer, head louse. *Pediculus vestimenti* (Linne) Nitzsch, body louse. *Plithirius inguinalis*, Redi., crab louse, *Dermacentor americanus*, Linn.; the hunting dog tick of the east is a pest to the hunter also *Dermacentor occidentalis*, Marx, MS. This Californian species, the one ordinarily found on deer; cattle and dogs was quite annoying to me during the past season. They get on one while passing through the chaparal and arriving at the base of the hair insidiously insert their beak into the skin. *Amblyomma unipunctata*, Packard, the "lone star" tick, so dubbed by a facetious Texan friend, from the iridescent spot on its back; is quite an annoying pest to those whose duties take them into the fields when it abounds *Ixodes scapularis*, Say, a tick with a brown shield and white body, frequent in the north and east. *Sarcoptes communis*, de Geer var hominis, the itch mite. *Demodex folliculorum*, Owen, the face mite; Packard's guide.

Nose: *Cephenomyia* (*sp. ignota*) The grub of this fly has been reported as being found in the nasal chambers of man by Riley and Howard Insect Life, vol. II, p. 116, 1889-90. This case as well as the reported case of *Cephenomgia* in hogs, Op. cit., vol. III, No.

vember, 1890, are beyond doubt cases of screw worm, *Lucilia macellaria.*

Intestine: *Ascaris lumbricoides*, Linn , the large round worm, *Trichocephalus dispar*, Rud., cæcum. Dr. D. S. Lamb, of the Army Medical Museum, writes me that he has seen this species in the human subject several times. The first time was in a girl five or six years old. Also in collection of Dr. G N. Acker, Washington, D. C. *Oxyuris vermicularis*, Bremser, pin worms or scab worms of the rectum. *Taenia solium*, Linn.; this species of tape worm is rare in collections. I have seen it in the collection of the Army Medical Museum and of Dr. J. S. Cram, of 5 N. Eleventh street, Philadelphia It is acquired by eating the young stage sequestered in measly or infested pork. *Taenia saginata*, Goeze, is by far the most common tape worm in the United States. Its young stage is found in beef. *Taenia flavopunctata*, Weinland; *Hy menoecpis dimnuta*, Rud., the original of the description occurred in this country. The *Synonym* is suggested by Dr. Stiles. *Bothricephalus latus*, Bremser. I have seen specimens from two patients. One specimen was donated to me by Dr. G. N. Acker, who obtained it from a Russian patient. The other is in the Army Medical Museum. It was taken from a Wisconsin soldier in the army of the Tennessee. From the rarity of its occurrence, I think that this species is not found in this country only as it appears now and then in people lately from Europe.

Abdomen, muscles, etc.: *Taenia solium*, Linn, in cystic stages scattered throughout the muscles. *Taenia acanthotrias*, Weinland, obtained once in Virginia and furnished the specimens for the original description (Verrill). *Taenia echinococcus*, v. Siebold, causes large tumors, especially in the liver, lungs, spleen, etc. Specimens Nos. 8,089, 8,792, 8,793 and 8,794 in the Army Medical Museum. Reported occasionally in medical journals. The adults of this species live in dogs. *Trichina spiralis*, Owen, scattering outbreaks due to the migrations of this well-known worm may be reported in the fall. Not common.

There has been no attempt made in the above lists to include the many biting or blood-sucking gnats and flies which are so common in summer time. Perhaps the well-known " buffalo gnat,' *Simulium pecuarum*, Riley, which often proves a fatal scourge to the cattle, horses and even man in the Mississippi bottoms ; the black gnats of the north woods, those pests of the hunters ; the many *abanidae*, those large flies whose piercing bite nearly drive the animals frantic, in summer and autumn ; the musquito specie of *Culex*, which trill their tiny song into our ears of a night-time, and all others of those pests who make it their duty to draw upon us or some one of the domesticated animals at sight for their fill of blood, should have been included ; but to make a complete list of those already known would consume more time than I can at present devote.

If the list, as presented above, fulfills the purpose of presenting to the student the majority of parasites actually seen in this country, my task is completed. It is for the future investigator, working on the material presented as a basis to amend names, to add omissions and record new species, thus artistically building up and finishing the pioneer work begun.

